# Controlled and Real-Life Investigation of Optical Tracking Sensors in Smart Glasses for Monitoring Eating Behavior Using Deep Learning: Cross-Sectional Study

**DOI:** 10.2196/59469

**Published:** 2024-09-26

**Authors:** Simon Stankoski, Ivana Kiprijanovska, Martin Gjoreski, Filip Panchevski, Borjan Sazdov, Bojan Sofronievski, Andrew Cleal, Mohsen Fatoorechi, Charles Nduka, Hristijan Gjoreski

**Affiliations:** 1 Emteq Ltd. Brighton United Kingdom; 2 Faculty of Informatics, Università della Svizzera Italiana Lugano Switzerland; 3 Faculty of Electrical Engineering and Information Technologies Saints Cyril and Methodius University in Skopje Skopje the Former Yugoslav Republic of Macedonia

**Keywords:** chewing detection, eating detection, smart glasses, automatic dietary monitoring, eating behavior

## Abstract

**Background:**

The increasing prevalence of obesity necessitates innovative approaches to better understand this health crisis, particularly given its strong connection to chronic diseases such as diabetes, cancer, and cardiovascular conditions. Monitoring dietary behavior is crucial for designing effective interventions that help decrease obesity prevalence and promote healthy lifestyles. However, traditional dietary tracking methods are limited by participant burden and recall bias. Exploring microlevel eating activities, such as meal duration and chewing frequency, in addition to eating episodes, is crucial due to their substantial relation to obesity and disease risk.

**Objective:**

The primary objective of the study was to develop an accurate and noninvasive system for automatically monitoring eating and chewing activities using sensor-equipped smart glasses. The system distinguishes chewing from other facial activities, such as speaking and teeth clenching. The secondary objective was to evaluate the system’s performance on unseen test users using a combination of laboratory-controlled and real-life user studies. Unlike state-of-the-art studies that focus on detecting full eating episodes, our approach provides a more granular analysis by specifically detecting chewing segments within each eating episode.

**Methods:**

The study uses OCO optical sensors embedded in smart glasses to monitor facial muscle activations related to eating and chewing activities. The sensors measure relative movements on the skin’s surface in 2 dimensions (X and Y). Data from these sensors are analyzed using deep learning (DL) to distinguish chewing from other facial activities. To address the temporal dependence between chewing events in real life, we integrate a hidden Markov model as an additional component that analyzes the output from the DL model.

**Results:**

Statistical tests of mean sensor activations revealed statistically significant differences across all 6 comparison pairs (*P*<.001) involving 2 sensors (cheeks and temple) and 3 facial activities (eating, clenching, and speaking). These results demonstrate the sensitivity of the sensor data. Furthermore, the convolutional long short-term memory model, which is a combination of convolutional and long short-term memory neural networks, emerged as the best-performing DL model for chewing detection. In controlled laboratory settings, the model achieved an *F*_1_-score of 0.91, demonstrating robust performance. In real-life scenarios, the system demonstrated high precision (0.95) and recall (0.82) for detecting eating segments. The chewing rates and the number of chews evaluated in the real-life study showed consistency with expected real-life eating behaviors.

**Conclusions:**

The study represents a substantial advancement in dietary monitoring and health technology. By providing a reliable and noninvasive method for tracking eating behavior, it has the potential to revolutionize how dietary data are collected and used. This could lead to more effective health interventions and a better understanding of the factors influencing eating habits and their health implications.

## Introduction

### Background

Obesity is a public health issue that leads to chronic diseases [1], including diabetes [2], cancer [3], and cardiovascular diseases [4]. In the United Kingdom, the obesity levels increased from 15% in 1993 to 28% in 2019 [5]. Similarly, in the United States, the obesity levels increased from 14.5% in 1970s to 39.6% [6]. Furthermore, poor diet was estimated to have contributed to 11 million deaths globally in 2017 [7].

Given these alarming statistics, gaining insight into people’s dietary habits is crucial for designing effective interventions aimed at promoting a healthy lifestyle. Dietary behavior tracking includes a spectrum of approaches ranging from manual to highly automated methods. At the most manual end, traditional food diaries require users to write down manually or digitally every item they eat or drink. The most commonly used manual tools to assess dietary intake and eating behaviors are 24-hour recalls, food records (food diaries), and food frequency questionnaires [8,9]. Major limitations of these methods include participant burden and recall or memory bias [10], which can lead to under- and overreporting of dietary intake. Digital tools and apps (eg, MyFitnessPal [11]) simplify the manual input process and integrate nutritional data, yet they require active user engagement, and in some cases nutrition knowledge to estimate calorie intake from precooked meals. A visual and less structured alternative is photographing meals, which offers an alternative way to recall and review dietary choices, sometimes shared with a dietitian for professional advice.

### Related Works

The existing research indicates a growing interest in developing automated tools for monitoring eating activities. Regarding the tools and studies closest to our study, several studies have explored monitoring eating activities based on sensor-enabled glasses. Most of these studies are focused on detecting eating or drinking episodes [12-16] and are performed in controlled environments [17,18]. Only one study has explored a more complicated scenario than the typical eating or noneating detection [18] by exploring the detection of chewing events using eyeglasses equipped with electromyography sensors in a study involving 10 participants both in controlled and in real-life conditions. Compared to the existing work, we present the first study to use smart glasses with integrated optical surface tracking sensors and deep learning (DL) to accurately identify both eating and chewing events, assessed both in controlled laboratory settings and through real-life trials, thus addressing research gaps and proving its efficacy in natural environments.

### Objective

This study aimed to develop and evaluate a novel, noninvasive system for automatically monitoring eating behavior by detecting eating and chewing activities. The system aims to enhance the accuracy and ease of tracking eating behaviors, addressing the limitations of self-reporting by providing precise, objective data.

The study provides a comprehensive evaluation of the proposed method using a combination of laboratory-controlled and real-life user studies, ensuring robust and noninvasive way to distinguish chewing activity from other activities, such as speaking, teeth clenching, grinding, smiling, frowning, brow raise, and winking.

The real-life data collection and analysis addresses a substantial gap in previous research and allows for the evaluation of the system’s performance in natural settings, providing insights into its practical application and adaptability.

## Methods

### Terminology

Throughout this study, several terms related to eating behaviors are used. To ensure clarity and consistency in their use, the following definitions are provided:

- Bite: the act of placing food into the mouth, chewing it, and then swallowing it as part of the eating process.
- Chew: a masticatory cycle involving the grinding or crushing of food with the teeth, preparing it for swallowing.
- Chewing: the overall process of breaking down food with the teeth.
- Chewing rate: the frequency of masticatory cycles (chews) per unit of time, measured in chews per second.
- Eating segment: a continuous period during which the participant consumes food without interruption, encompassing consecutive bites and chewing cycles without pauses between bites. Thus, one eating segment can include one or several bites and chewing events.

### Smart Glasses and Data Collection Setup

#### Overview

In this section, we describe our data collection setup, providing insights into the configuration and sensors of the used smart glasses. In addition, we describe the methodologies used for data collection in both controlled laboratory settings and real-life scenarios.

In contrast to the methods that require manual input, in this study, we propose an approach to automatic monitoring of eating behavior by monitoring facial muscle activations using optical sensors incorporated in smart glasses frame. The approach offers real-time feedback that can be integrated with mobile health apps, allowing users to monitor their dietary habits seamlessly. The data collected can be used to personalize dietary recommendations, support weight management programs, and contribute to research in nutritional epidemiology. Ultimately, the goal is to empower individuals with actionable insights to improve their eating habits and promote long-term health and well-being.

**Figure 1:**
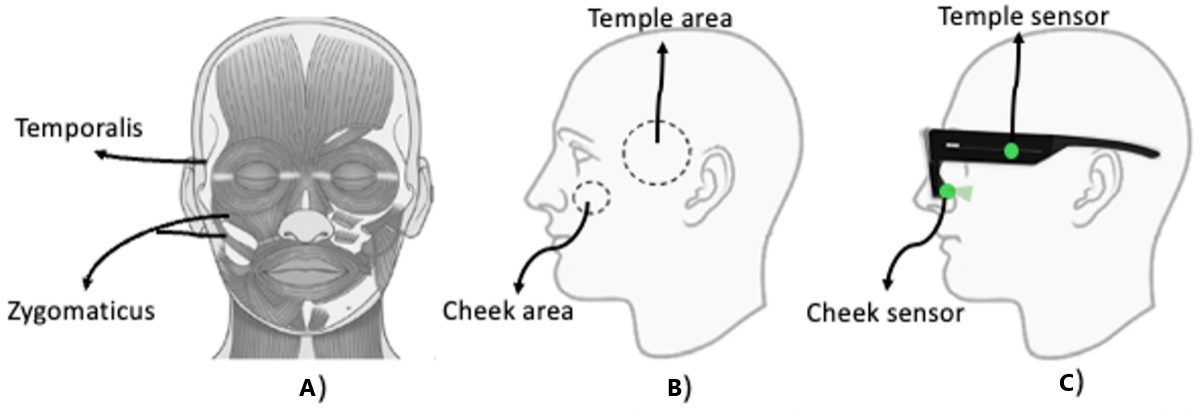
(A) The 2 types of facial muscles related to chewing (temporalis and zygomaticus); (B) monitored skin areas by the smart glasses; and (C) the placement of OCO sensors within the glasses frame.

The proposed system is depicted in Figure 1: (A) facial muscles associated with chewing that we aim to monitor; (B) the areas of skin that are monitored by the system; and (C) OCO optical sensors embedded in smart glasses. One of the muscles associated with the chewing activity is the temporalis muscle. The temporalis muscle is near the temple and extends downward in a direction toward the mouth. It controls movement of the lower jaw (eg, opening and closing of the mouth). This area is monitored by the OCO *temple* sensor in the glasses. Other muscles that are activated during chewing are the cheek muscles such as zygomaticus major and minor. This area is monitored by the OCO *cheek* sensor in the glasses. Our approach is based on the assumption that chewing activates multiple facial muscles, which causes the facial skin to move in a parallel direction relative to the sensors embedded within a glasses frame. These movements of the facial skin in the X-Y plane are monitored by our novel patented optical tracking sensors—OCO. The optical sensor data are then analyzed using DL to distinguish chewing activity from other activities that cause facial skin movements, such as speaking, teeth clenching, smiling, frowning, winking, and similar.

#### OCOsense Smart Glasses and OCO Sensors Data

The OCOsense smart glasses integrate 6 optical tracking—OCO sensors [19], 3 proximity sensors, a 9-axis inertial measurement unit, an altimeter, and dual speech detection microphones. The OCO sensors use optomyography, an optical noncontact methodology, to measure skin movement in 2 dimensions resulting from underlying myogenic activity. They consist of an optical surface tracking sensor that measure relative movements on the skin’s surface in 2 dimensions (*X and Y* dimensions). These sensors operate accurately within a range of 4 to 30 mm without requiring direct skin contact [19]. Positioned within the glasses frame, their focus lies on monitoring skin movement over specific facial muscle groups, including the frontalis and corrugator muscles on both sides of the forehead, the zygomaticus major and minor muscles on the left and right sides of the cheeks, the orbicularis muscles around each eye, and the left and right temples.

The eating activity activates two types of facial muscles that we can monitor with the glasses: (1) the temporalis muscle, which is near the temple, and controls movement of the lower jaw (opening and closing of the mouth), and (2) zygomaticus major and minor, which are located in the cheek area, and are activated during the chewing activity. Therefore, in this paper, we primarily focus on data collected from the cheek and temple OCO sensors (marked with green rectangles in Figure S1 in Multimedia Appendix 1), as these areas are more relevant to eating activity, compared with the rest of the sensors available in the glasses (marked with red rectangles in Figure S1 in Multimedia Appendix 1). A corresponding sensor data are presented in Figures S2 and S3 in Multimedia Appendix 1.

#### Data Collection Methodology

For development and evaluation of our method we collected 2 data sets. The first data set was collected in laboratory environment, while the second data set was collected in-the-wild. The laboratory data enabled us to establish a foundational understanding of eating behaviors under controlled conditions. However, evaluating the method on real-life data allows for assessing its generalization capability and adaptability to diverse and unpredictable environments.

To be more precise, we used the laboratory data sets to:

- Perform a statistical analysis comparing measurements obtained during 3 activities (eating or chewing, speaking, and teeth clenching) from both temple and cheek sensors, assessing skin movement along both the X and Y axes. This analysis is based on data from 28 participants for whom we have both eating and noneating labeled data.
- Develop and evaluate DL models for chewing detection. We compared the performance of four DL architectures. For the best-performing DL method, we conducted a more detailed analysis, including the impact of individual sensors on the performance of DL models (eg, temple, cheek, temple + cheek, left temple + left cheek, and right temple + right cheek), and the impact of segmentation window size on the performance the DL models, varying the window size between 2 and 15 seconds.

We used the real-life data set to evaluate chewing detection and eating segments detection methods using data collected in-the-wild. Summary of the collected data sets is presented in Table 1.

Participant recruitment involved booking a time for data collection through social media announcements and completing a Google form to confirm eligibility. Eligible participants were required to be in good health; with no history of eating disorders; and without dietary restrictions, allergies, or intolerances. In addition, participants with conditions affecting facial muscle activation, such as stroke or facial palsy, or any other conditions impacting normal and symmetrical chewing and swallowing were excluded. An important inclusion criterion was that participants have proper glasses fit to ensure accurate detection of skin movements by the sensors.

**Table 1:** Summary of collected data sets.

Data set	Participants, n	Median duration	Total duration
Eating (laboratory)	28	9 min 49 s	369 min 15 s
Noneating (laboratory)	126 (same 28+98 new)	11 min 50 s	1601 min 47 s
Real life	8	907 min	7163 min

#### Study Procedures

##### Laboratory-Based Data Collection (Controlled Environment)

In the laboratory-based experiments, we collected two data sets:

1. Eating data set: the participants engaged in a full meal, providing them with the freedom to choose from a diverse range of food options, including:
  Crispy or hard foods: apples, carrots, nuts, crisps, and crackers
  Creamy or soft foods: porridge, banana, yogurt, fruit salad, and green salad
  Chewy foods: breakfast bars; pop-tart; toast, bagel, or croissant; and biscuits

In addition, they were allowed to eat with or without utensils, based on their preference. There were no time constraints for completing the meal. Participants ate their meals in a laboratory setting designed to simulate a natural dining environment. They consumed their meals alongside the researchers, which helped create a more relaxed and realistic atmosphere. Despite the laboratory setting, participants were encouraged to consume their meals in a natural manner, simulating real-life conditions. This allowed for varied behaviors, for example, some participants used their phones during meals and others engaged in conversations. During the data collection, the participants were continuously video recorded, providing synchronized data between the video recording and sensor data. This enabled us to label each chewing segment later manually. For the eating activity, we annotated all segments where the participants had food in their mouth. Two researchers independently coded the bites, ensuring reliability and validity through cross-verification. The availability of video data allowed for accurate annotation, as each segment was reviewed by at least 2 researchers to confirm the presence of food in the participants’ mouths.

2. Noneating data set: the data collection was performed in a controlled laboratory setting, where participants were instructed on the activities they should perform. First, participants performed a subset of activities associated with facial muscle engagement. This category includes brushing teeth, engaging in conversation, reading aloud, and diverse expressions of bruxism, encompassing teeth clenching, grinding, and tapping. In addition, we incorporated various facial expressions and gestures, such as smiling, frowning, winking, and similar, to capture a diverse range of facial movements. Moreover, we included a variety of activities that do not specifically rely on facial muscle engagement. These include hygiene-related activities such as handwashing and dishwashing, routine activities such as walking and sitting in a chair, and physical activities such as jogging and stair climbing.

##### Real-Life Data Collection (Uncontrolled Environment)

In the real-life setting, the participants were instructed to wear the OCOsense smart glasses continuously for a minimum of 8 hours a day over a span of 2 days. The participants were allowed to follow their daily routines without any imposed limitations during this period. This enabled the capture of eating behaviors in various settings such as home, workplace, and other public spaces. In addition, there were no restrictions placed on participants regarding their food choices or other diet-related decision. For the data collection procedure, we developed an application that collects data from the glasses and enables the participants to annotate when engaged in eating activities. More specifically, they were asked to press a button when they start eating and press it again when they finish eating. A researcher monitored the number of labeled eating events per day per participant. In instances where participants forgot to press the start or end buttons, they were asked to note the approximate times of their eating sessions. These cases were then manually analyzed by a researcher using the sensor data from the glasses to provide precise labels for the eating start and end times. These labeled segments served as the ground truth for subsequent experiments. The annotations collected with this approach result in whole data segments labeled as eating, yet these segments may also include a range of activities beyond eating itself, such as engaging in conversation or pausing briefly between bites, which typically occur during regular real-life meals.

### Statistical Analysis

#### Data Preprocessing

To perform statistical comparison, the following data preprocessing steps were applied to the sensor data:

1. Calculation of the vector magnitude for each sensor: as the OCO sensors measure skin movement in 2 dimensions (*X* and *Y*), the vector magnitude was calculated for each sensor (
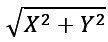
).
2. Combination of processed sensor signals values from the left and right sensors: the vector magnitude value from the left cheek sensor was added to the vector magnitude value from the right cheek sensor, and the same was done for the temple sensors. This resulted in the creation of 2 signals, one representing the total cheek movement (left+right), and one representing the total temple movement (left+right).
3. Smoothing of the resulting signals: the resulting cheek and temple signals were smoothed using a rolling median filter with a window size of 15 samples (0.3 s) to reduce the effects of noise on the signals.

#### Hypothesis Testing

Hypothesis testing was conducted using the Wilcoxon signed-rank test, a nonparametric alternative to the paired 2-tailed *t* test. This test evaluates the distribution of differences between related paired samples to ascertain whether they originate from the same distribution. The null hypothesis is that the samples derive from the same distribution. To account for multiple comparisons, *P* values were adjusted using the Bonferroni correction method (α=.05).

### Chewing Detection Methodology

#### Overview

**Figure 2:**
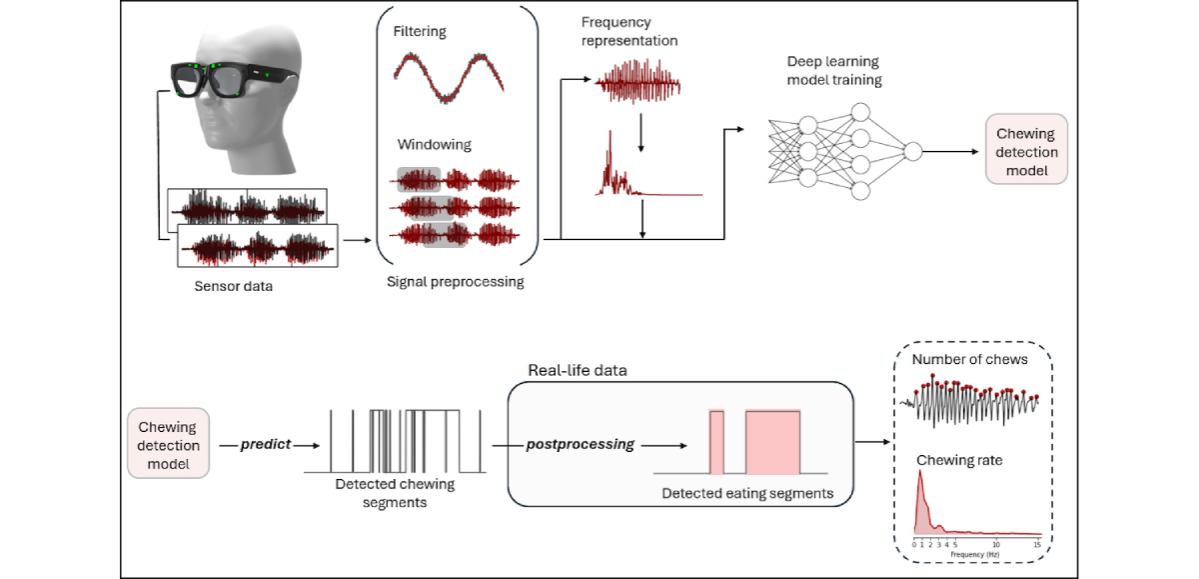
Overview of the developed method for detection of chewing and eating segments estimation.

This section describes the method used in this study for automatic chewing and eating segment detection. Initially, the sensor data undergoes preprocessing, including filtering and segmentation into windows. Then, both the filtered signals and their frequency representations are used as input to DL models, which classify the windows into chewing or nonchewing. To enhance the accuracy in real-life scenarios, we introduce a supplementary model—hidden Markov model (HMM). This integration enables the grouping of chewing predictions and the construction of coherent eating segments. Finally, we calculate the number of chews and the chewing rate for the detected eating segments. The block diagram of the pipeline is shown in Figure 2.

#### Signal Preprocessing

The method uses data from the 4 OCO sensors—left and right temple and left and right cheek. Let *O_RC_, O_LC_, O_RT_, and O_LT_* denote these sensors in the specified sequence. The set of sensors can be represented as S = {*O_RC_, O_LC_, O_RT_, O_LT_*}, where each sensor *S^i^* reads data 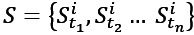. in the time interval of *T* from timestamps *t_1_* to *t_n_*. The main objective is defined as:

1. Partitioning *T* into partially overlapping windows of equal size *W* = *{W_1_,W_2_,...,W_n_}* and assuming a target activity set *Y* = *{Y_1_,Y_2_,...,Y_n_}*
2. Assigning each window *W_i_* a target label *Y_j_* from the target label set *Y* = *{Y_1_,Y_2_,...,Y_n_}* and training a classifier accordingly

First, to remove the noise from the data, a fifth order median filter was applied to each sensor channel within the sensor set *S*. This filter was proven to effectively remove noise while preserving essential signal features in our previous studies on expression recognition using the same type of sensor [20]. Following the medial filter, the next step in the process involved determining the appropriate window size for data segmentation. We experimented with various window sizes ranging between 1 and 15 seconds. Once the sensor set *S* was segmented into windows (*W*), the next step was to enhance the information carried by the input signals further. To achieve this, we used Fourier transformation for each sensor within the segmented windows. This transformation allowed us to convert the time-domain signals into their frequency representations, thereby extracting additional features from the data. The Fourier transformation process provided valuable insights into the frequency components present in the sensor data, which could be crucial for detecting subtle patterns associated with chewing activity.

#### Chewing Detection With DL Models

In this study, we used 4 distinct DL models based on convolutional neural networks (CNNs) for the purpose of chewing detection. We focused on DL architectures commonly used for wearable sensor data, such as CNN 1D [21,22], CNN 2D [21], attention model [23], and convolutional long short-term memory (ConvLSTM) [24]. By using these common architectures, we aim to demonstrate the baseline accuracy achievable with existing methods. This serves as a foundation upon which further improvements can be made. Specifically, developing DL architectures tailored to the unique specifications of the glasses and the specific use-case of detecting eating activity could potentially enhance accuracy beyond the baseline results established in this study.

An overview of the architectures and their associated hyperparameters is as follows:

1. CNN 2D: Our initial model adopts a standard CNN [21], crafted to extract hierarchical spatial features from input data. The feature extraction module consists of 3 consecutive convolutional layers, each followed by group normalization and max-pooling layers. Extracted features are then passed through 2 fully connected layers, each containing 128 neurons, connecting to the output nodes.
2. ConvLSTM: Expanding on CNN’s foundation, the ConvLSTM model [24] introduces a temporal dimension to our analysis. It shares the same convolutional layers with the CNN 2D architecture and integrates 2 LSTM layers, each featuring 128 hidden units. This modification allows the model to effectively capture sequential patterns and dependencies within the data.
3. Attention model: Incorporating insights from attention mechanisms, the attention model [25] comprises 4 convolutional layers with 64 feature maps followed by 2 LSTM layers, each with 128 hidden units [23], and an attention layer. The attention layers allow the model to prioritize relevant information during the learning process.
4. CNN 1D with statistical features: The last model incorporates a 1D CNN architecture [22], enhanced with statistical features. It consists of a single convolutional layer with 256 filters followed by a max-pooling layer. The resulting features are then flattened and fused with statistical features extracted from filtered sensor data, including mean, variance, and absolute sum. The joint vector is then processed through a fully connected layer with 1024 neurons capturing both spatial and statistical characteristics.

The determination of architecture parameters, such the kernel size in the convolutional layers, output size of CNN layers, LSTM units, and fully connected units, was guided by a pragmatic approach focused on achieving a balance between model’s ability to capture complex data patterns and model’s complexity. These parameters were fine-tuned on the validation set to optimize performance.

Each model was trained for 100 epochs with a batch size set at 256. Prior the beginning of the learning process, we used orthogonal weight initialization for both weights and biases, aiming to enhance the stability and effectiveness of neural network training. Cross-entropy loss was used as the objective function for training. Furthermore, all the models were trained using the Adam optimizer with an initial learning rate of 1e-3. To avoid overfitting as well as to reduce the training time, early stopping, monitoring validation *F*_1_-macro score with patience of 15 epochs was applied. In the end, the optimal weights were selected based on the epoch with the highest validation *F*_1_-macro score.

#### Detection of Eating Segments

In the initial phase of our eating detection system, we use a DL model to detect chewing moments at a window-level granularity. By incorporating the temporal dependence between the detected chews, we aim to enable our system to identify not only individual chewing instances but also to discern when eating segments occur within real-life data. This allows us to effectively mitigate the occurrence of short false-positive predictions and consolidate densely clustered chewing instances into coherent eating segments. By doing so, we anticipate a more robust and precise analysis of dietary patterns.

To address the temporal dependence between chewing events in real life, we integrate HMM as a supplementary model that analyzes the detected chews from the DL model. The HMM was initialized and trained as described in the study by Stankoski et al [26]. This process is visually illustrated in Figure S4 in Multimedia Appendix 1.

#### Detection of Number of Chews and Chewing Rate Estimation

**Figure 3:**
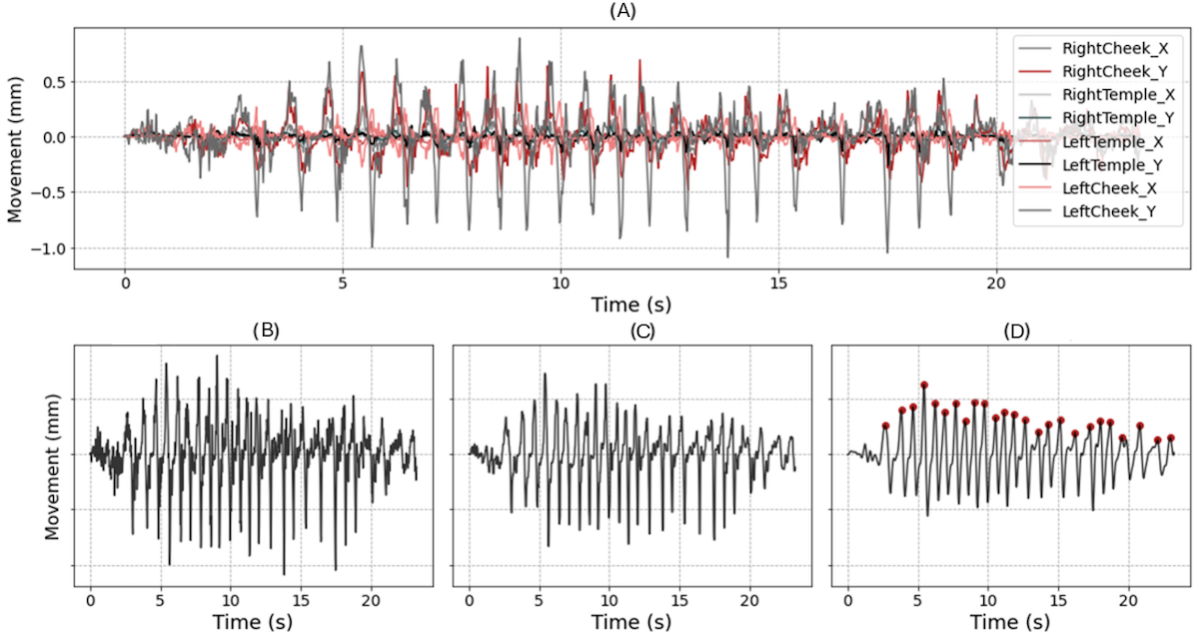
Processing steps for detecting the number of chews in an eating segment: (A) cheek and temple signals; (B) selection of signal with highest root mean square (RMS) value; (C) filtering the chosen signal; (D) detection of peaks and chews in the filtered signal.

After the detection of eating segments, to determine the number of chews in an eating segment, we additionally analyze and process the signals from the sensors. Figure 3 presents a visual representation of the data processing steps used in the detection of chews within a randomly selected eating segment from the data set. The initial step involves identifying the signal with the highest root mean square value. Subsequently, we use a 2-step filtering process to enhance the selected signal. First, a median filter with a kernel size of 5 is applied, followed by a second-order bandpass filter within the frequency range of 0.5 to 3 Hz. For the calculation of the number of chews, we used an existing peak detection algorithm (using SciPy [27]) on the processed signal. This involves configuring the threshold and distance parameters to identify relevant peaks in the signal. Furthermore, peaks with insufficient prominence are excluded from the final set.

Each retained peak after this step is considered as a separate chew in the signal. The parameter values used in the filtering and peak detection processes were determined empirically.

Following the detection of chews within eating segments, we extend the analysis to estimate the chewing rate. To achieve this, we use the same signal with the highest root mean square value. This signal is subjected to further analysis through Fourier transformation to compute its frequency spectrum. By examining the resulting spectrum, we identify the most substantial frequency component, which corresponds to the dominant chewing frequency within the examined eating segment.

### Evaluation Setup

To evaluate the effectiveness of the models, we used the Leave-One-Group-Out cross-validation technique. This involved dividing the initial data set into N separate groups, where the data from a single participant is present in only one subset. Each model is trained on combined data from *N*-2 subsets, leaving one subset to be used as validation data set and a second subset for testing the final model. Thus, all the models are person-independent, that is, the experimental results demonstrate the model’s accuracy on unseen test users.

Regarding evaluation metrics, we used recall, precision, and *F*_1_-score. Recall indicates the proportion of actual chewing segments correctly identified by the model, while precision denotes the proportion of identified chewing segments that are truly chewing segments. The *F*_1_-score is the harmonic mean of the recall and the precision—which is more balanced metric compared with accuracy especially in unbalanced data sets where one of the classes is more frequent. The reported metrics reflect the models’ ability to detect chewing at a window level, and they are calculated as follows:



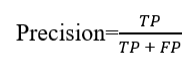



**(1)**



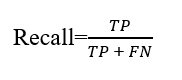



**(2)**



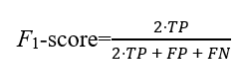



**(3)**

In the equations (1) to (3), *TP* represents true positives, *TN* represents true negatives, *FP* represents false positives, and *FN* represents false negatives. In the context of chewing detection, these metrics can be interpreted as follows:

- *TP* indicates the number of windows from the chewing class correctly classified as chewing.
- *FP* indicates the number of windows from the nonchewing class incorrectly classified as chewing.
- *FN* indicates the number of windows from the chewing class incorrectly classified as nonchewing.

In addition, for the evaluation of eating detection in the real-life scenario, we used custom metric to provide deeper insights into the models’ performance within eating segments. The metric was defined to analyze the number of eating segments that are correctly identified based on the frequency of positive chewing predictions within each eating segment:

- Detected eating segments: the number of eating segments where at least 50% of the instances (windows) are correctly identified as eating.

### Ethical Considerations

To ensure ethical compliance, ethics approval was obtained from the London—Riverside Research Ethics Committee on July 15, 2022 (ref: 22/LIO/0415). After a detailed explanation of the experimental procedure, all participants provided written informed consent before participating in the study. The consent forms addressed the use of their data. To protect participant privacy, all data were deidentified. The participants who took part in the laboratory sessions were compensated with US $26.5, while those involved in the real-life study received US $26.5 per day for their participation. The experiment was conducted following institutional ethical provisions and the Declaration of Helsinki.

## Results

### Overview

In this section, we present the results from the experiments. The *Statistical Analysis of OCO Sensors for Facial Muscle Movements* section presents the outcomes of the statistical analysis, focusing on the ability of OCO sensors in detecting facial muscle movements during various activities, including eating. The *Laboratory-Based Data Set DL Experiments* section assesses the performance of different DL models, sensor combinations, and window sizes for chewing detection in a controlled laboratory data set. Finally, the *Real-Life Data Set Experiments—Chewing and Eating Segments Detection* section presents the results obtained with the real-life data set and evaluate the performance of the method for detection of eating segments.

### Statistical Analysis of OCO Sensors for Facial Muscle Movements

**Figure 4:**
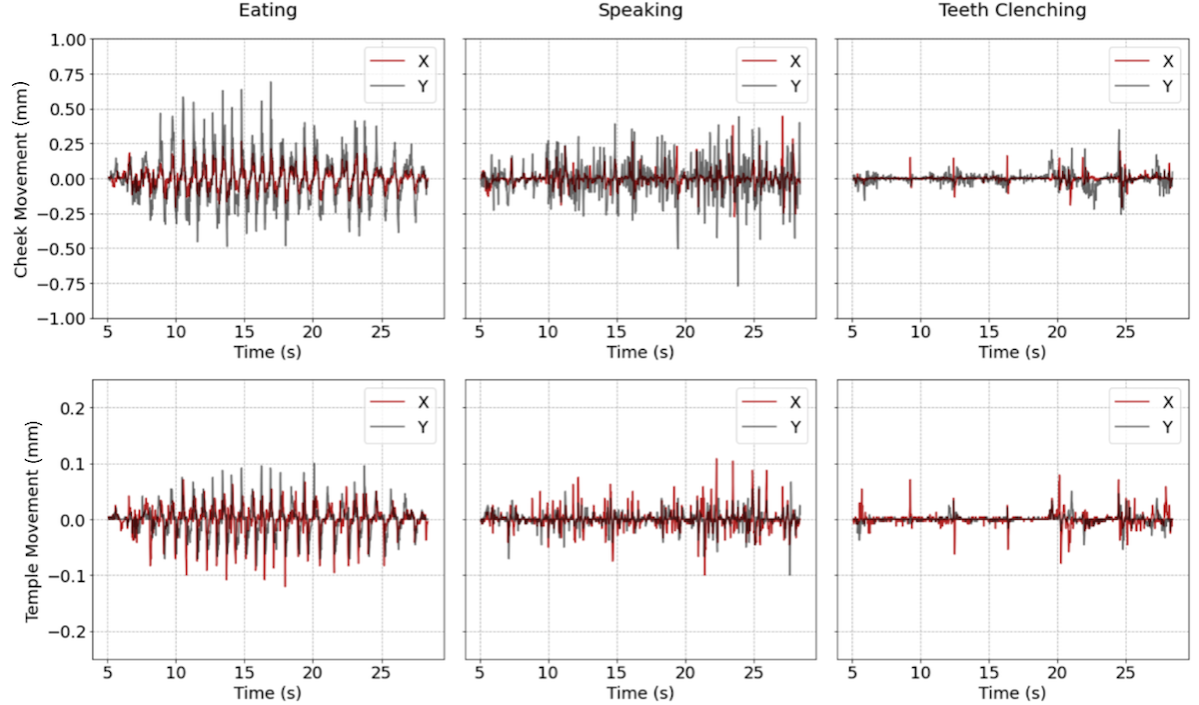
Sensor signals from the sensors on the right cheek and temple recorded during eating, speaking, and teeth clenching activity performed by one participant.

To evaluate the ability of the OCO sensors to detect facial muscle movements during different activities, we first conducted a statistical analysis. Our focus was on comparing measurements obtained from both temple and cheek sensors, assessing skin movement along both the *x* and *y* axes. In this context, we focused on comparing facial muscle movements during the activities of eating or chewing, speaking, and teeth clenching. The selection of these activities was based on the potential similarity in facial muscle activation patterns. For example, Figure 4 presents 6 graphs. The top row measures movements from sensors placed over the zygomaticus major muscle (cheek area) and the bottom row from sensors positioned on the temples. Each column of graphs represents 1 of the 3 activities being measured (eating, speaking, and clenching). The horizontal axis of each graph represents time in seconds, and the vertical axis shows the magnitude of skin movement in millimeters. By comparing these graphs, we can assess the differences and similarities in facial muscle activation patterns during the 3 activities.

We calculated mean movements measured from the cheek and temple OCO sensors for each participant during eating or chewing, speaking, and teeth clenching. The mean values were calculated over all data points corresponding to each activity, resulting in n=28 (number of participants present in both the eating and noneating laboratory data set) tuples, with each tuple comprising 3 values representing the mean cheek or temple movement for eating or chewing, speaking, and teeth clenching.

**Figure 5:**
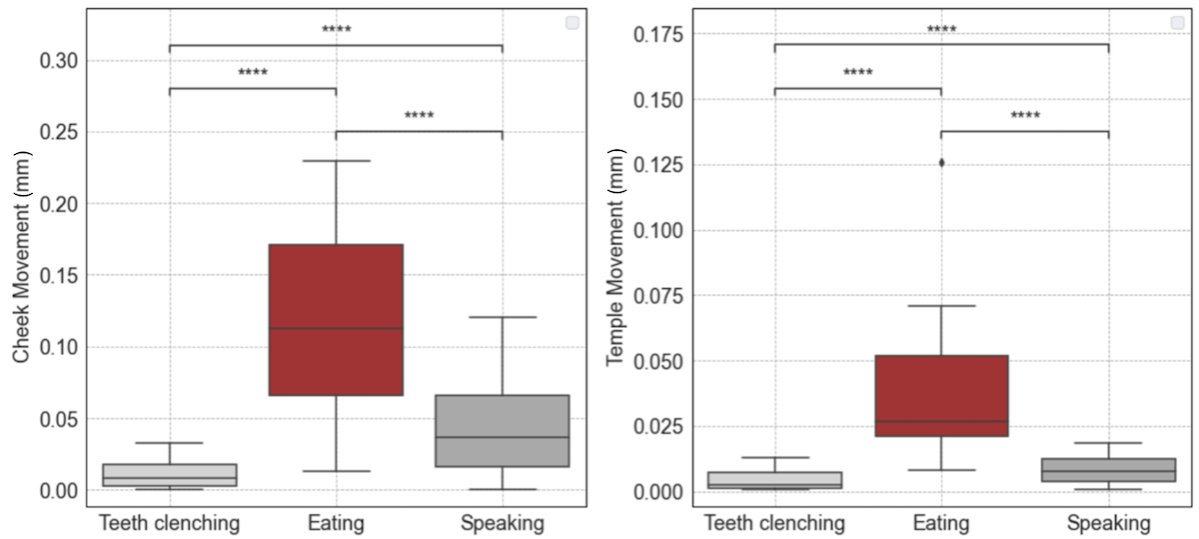
Wilcoxon signed-rank (paired) test with Bonferroni correction for comparing mean cheek and temple movements during activity pairs (n=28): clenching versus eating, clenching versus speaking, and eating versus speaking. Statistical significance annotations: *If P ∈ {.05, .01); **if P ∈ {.01, .001); ***if P ∈ {.001, .0001); and ****if P≤.001.

Figure 5 shows the mean cheek (left plot) and temple (right plot) movements during different activities, presented on the x-axis, and the results from the Wilcoxon signed-rank (paired) test with Bonferroni correction (α=.05).

For the cheek OCO sensors, we can observe an increased movement during eating (median value 0.113 mm) compared with relatively lower values observed during speaking (median value 0.036 mm) and the teeth clenching (median value 0.008 mm). The results from the statistical test further indicate significant differences in cheek movements between speaking and eating (*P*<.001), eating and teeth clenching (*P*<.001), as well as speaking and teeth clenching (*P*<.001).

Similarly, for the temple OCO sensors, a notable increase in movement with a median value of 0.027 mm during eating is observed, compared with 0.008 mm during speaking and 0.002 during the teeth clenching. The statistical tests affirm the significance of these differences, demonstrating that mean temple movements differ significantly between speaking and eating (*P*<.001), eating and the teeth clenching (*P*<.001), as well as speaking and the teeth clenching (*P*<.001).

These findings highlight the potential sensitivity of the cheek and temple OCO sensors in capturing distinct patterns and subtle variations in facial muscle activation across different activities.

### Laboratory-Based Data Set DL Experiments

In this section, we present the sample characteristics of the data set, the results of the experiments for chewing detection, conducted on the laboratory-based data set, offering insights into the results achieved across various DL architectures, sensor combinations, and window sizes.

#### Sample Characteristics

The laboratory-based data set consists of 2 subsets, one for eating activities and another for noneating-related activities. In the controlled eating data set, we gathered data from a cohort of 28 participants, comprising 13 (46%) males and 15 (54%) females, with an average age of 25.6 (SD 9.1) years. The data set comprises a total of 6.1 hours of recorded data. The noneating data set includes data from the same 28 participants, along with an additional 98 participants (n=48, 49% males and n=50, 51% females) with an average age of 23.3 (SD 6.4) years. Each participant contributed data for various activities, totaling 26.7 hours of recorded data. In summary, the data set comprises 126 participants and spans a combined total of 32.8 hours of recorded data.

#### DL Models for Chewing Detection

In this section, we present a comparison of various DL architectures used for the task of chewing detection. Table 2 provides a summary of the performance metrics, including *F*_1_‑score, recall, and precision for the chewing class, for each architecture.

The results show that all architectures demonstrated strong results, indicating that the sensor data provided from the glasses is informative for the chewing detection task. ConvLSTM demonstrated the highest *F*_1_-score of 0.91, precision of 0.92, and recall of 0.89. CNN 2D also performed well with balanced metrics, achieving a slightly lower precision of 0.90, recall of 0.90, and *F*_1_-score of 0.90. In contrast, the attention model displayed moderate performance with precision, recall, and *F*_1_‑score of 0.89, 0.90, and 0.89, respectively. The CNN 1D architecture, despite exhibiting a high precision of 0.90, fell short in recall at 0.86, resulting in a lower overall *F*_1_-score of 0.88.

**Figure 6:**
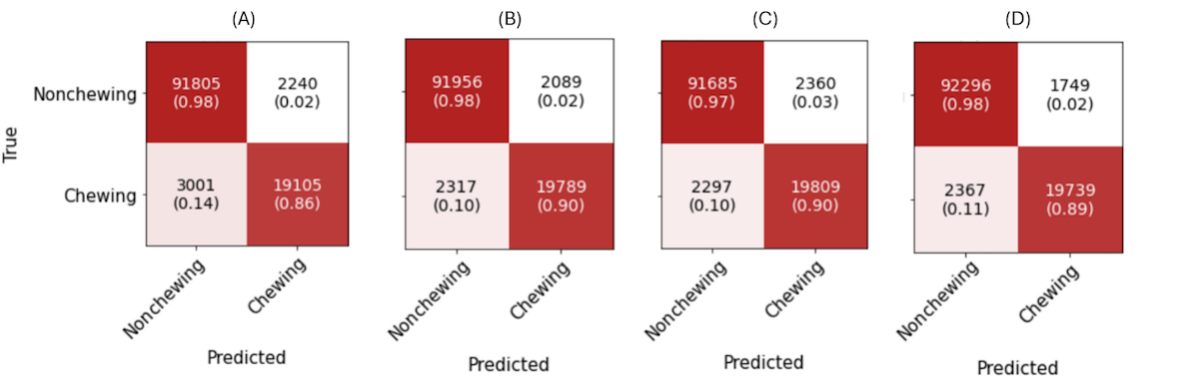
Confusion matrices for the evaluated deep learning architectures: (A) convolutional neural network (CNN) 1D; (B) CNN 2D; (C) attention model; (D) convolutional long short-term memory (ConvLSTM).

The confusion matrices for the evaluated models are presented in Figure 6. They provide additional insights into the models’ behavior. Notably, the ConvLSTM model also demonstrated a lower number of false positives (FPs), totaling 1749 instances. This number is approximately 20% lower than that of the second-best model, CNN 2D, which recorded 2089 FP instances.

Figure S5 in Multimedia Appendix 1 shows the FP rates for various noneating activities detected by the ConvLSTM model. Socializing has the highest rate (0.72%), followed by reading (0.28%). Both involve speaking, leading to confusion with eating due to similar facial movements. The overall FP rate is 2%, also shown in Figure 6 (0.02 in confusion matrix D).

Table 3 provides a comparison of complexity and resource metrics for the evaluated architectures focusing on network parameters, computational complexity expressed as the number of floating-point operations per second (FLOPS) during a forward pass and the size of the model. CNN 1D with 2.4 gigaFLOPS and the 270-kB model size is the smallest model making it suitable for embedded applications in the future. CNN 2D, although larger, offers a balanced trade-off between performance and model size. The attention model, despite having fewer parameters than CNN 2D, has the highest computational complexity of 80.25 gigaFLOPS. ConvLSTM demonstrates a balance between accuracy and resource requirements.

Considering the results, ConvLSTM emerged as the preferred choice for the chewing detection tasks based on the model accuracy and computational complexity and resources needed, thus this architecture was used in the subsequent experiments.

**Table 2:** Performance metrics of different deep learning architectures for chewing detection. Precision, recall, and F1-score are calculated for the eating class.

DL architecture	Precision	Recall	*F*_1_-score
CNN^a^ 1D	0.9	0.86	0.88
CNN 2D	0.9	0.9	0.9
Attention model	0.89	0.9	0.89
*ConvLSTM* ^b^	*0.92* ^c^	*0.89*	*0.91*

^a^CNN: convolutional neural network.

^b^ConvLSTM: convolutional long short-term memory.

^c^Best performing algorithm.

**Table 3:** Complexity and resource metrics for the evaluated deep learning (DL) architectures.

DL architecture	Total parameters	Computational complexity (GFLOPS^a^)	Model size (MB)
CNN^b^ 1D	43,366	2.41	0.27
CNN 2D	1,208,578	20.92	4.88
Attention model	407,620	80.25	2.68
ConvLSTM^c^	997,890	31.42	4.03

^a^GFLOPS: giga floating-point operations per second.

^b^CNN: convolutional neural network.

^c^ConvLSTM: convolutional long short-term memory.

#### Impact of Individual Sensors on the Performance of Chewing Detection Models

In this section, we present the results from the analysis of the impact of individual sensors on the performance of the chewing detection models. Having identified the ConvLSTM architecture as the best-performing architecture among the models that we evaluated in the previous experiments, we proceeded with this architecture for a series of experiments encompassing various sensor combinations. The tested sensor combinations included temple, cheek, temple and cheek, as well as the left versus right side. The results from these experiments are presented in Table 4.

From Table 4 it can be observed that the cheek sensor outperforms the temple sensor. Specifically, in detecting chewing segments, the cheek sensor achieves recall of 0.88, which is 4 percentage points higher than the recall achieved by the model trained with temple sensor data (0.84).

The performance of the model trained with data from the cheek sensors can be attributed to the role of the cheek region in eating activities, predominantly chewing. The sensors are adept at capturing the specific circular movements of the cheek area during such activities, which produce distinct signal pattern associated with eating. The ability to capture these specific patterns results in the model’s high precision in distinguishing eating episodes, thus enhancing recall rates.

Although the temple muscle is uniquely activated during chewing activity, the results show that the activation measured by the sensor is not very high across all people. However, if we combine the temple and the cheek sensors, we can see that the recall is improved by 1 percentage point. This shows that the temple sensor data provides additional information to the model.

In addition, we explored the performance of the models by using only one side of the temple and cheek sensors. On the basis of the results, we can see that the combination with the sensors measuring the right temple and cheek achieves recall of 0.89, which is 3 percentage points higher than the recall achieved by the model trained with left temple and cheek sensor data. This might be expected because most people prefer to chew the food on one side of their mouth [27,28] and the activation of the muscles is higher, which results in higher values in the sensor data.

**Table 4:** Performance metrics of convolutional long short-term memory for chewing detection with multiple combinations of sensor data. Precision, recall, and F1-score are calculated for the eating class.

Sensor combination	Precision	Recall	*F*_1_-score
Temple	0.83	0.84	0.83
Cheek	0.92	0.88	0.90
*Temple+cheek*	*0.92* ^a^	*0.89*	*0.91*
Left temple+left cheek	0.9	0.86	0.88
Right temple+right cheek	0.89	0.89	0.89

^a^The selected combination shows the best results based on the *F*_1_-score.

#### Window Size Impact on the Performance of Chewing Detection Models

This section presents the results of the analysis of how window size influences the performance of the chewing detection models. For this purpose, a series of experiments were conducted, exploring various window sizes that extend beyond the default 4-second window size used in the previous experiments. For this analysis, we used a consistent 1-second window slide, with the aim to prevent delays in prediction changes and to ensure that the model will be able to promptly detect eating-related movements. The results from the experiments are presented in Table 5.

The performance of the ConvLSTM architecture demonstrated a noticeable enhancement with the increase in window size in terms of precision, recall, and *F*_1_-score. More specifically, as the window size extends from 2 to 10 seconds, we consistently observe improvements in results. However, upon reaching a 15-second window, we observe saturation in performance metrics, where the obtained results remain consistent with those achieved at the 10-second window. This is probably because longer windows might include nonchewing data, leading the model to misclassify entire instances as noneating.

Although, among the window sizes of 6- and 10-second improvement can be observed, we decided to proceed with the 4-second window. This decision was based on its advantage in processing fewer data compared with the 6- and 10-second modes, leading to a reduced computational demand and potentially lower energy use.

**Table 5:** Performance metrics of convolutional long short-term memory for chewing detection with various window sizes. Precision, recall, and F1-score are calculated for the eating class.

Window size	Precision	Recall	*F*_1_-score
2 seconds	0.90	0.86	0.88
4 seconds	0.92	0.89	0.91
6 seconds	0.93	0.92	0.92
*10 seconds*	*0.93* ^a^	*0.94*	*0.93*
15 seconds	0.92	0.94	0.93

^a^Best performing result.

### Real-Life Data Set Experiments: Chewing and Eating Segments Detection

#### Overview

To assess the effectiveness of our chewing detection and eating segments detection methods using data collected in-the-wild, we conducted a series of experiments. In the first subsection, we present the sample size of the data set. Then, in the second subsection, we present the results of the chewing detection method using real-life data. Next, in the third subsection, evaluation of the eating segment detection is presented. In the last subsection, we show the estimation of the chewing characteristics.

#### Sample Characteristics

The real-life setting data collection involved 8 participants (5 males and 3 females; average age 30.8, SD 12.4 years). Each participant wore the glasses for a minimum of 8 hours per day over 2 days, resulting in 16 hours of recorded data per participant and a total of 128 hours of recorded data.

#### Chewing Detection Evaluation Using Real-Life Data

**Figure 7:**
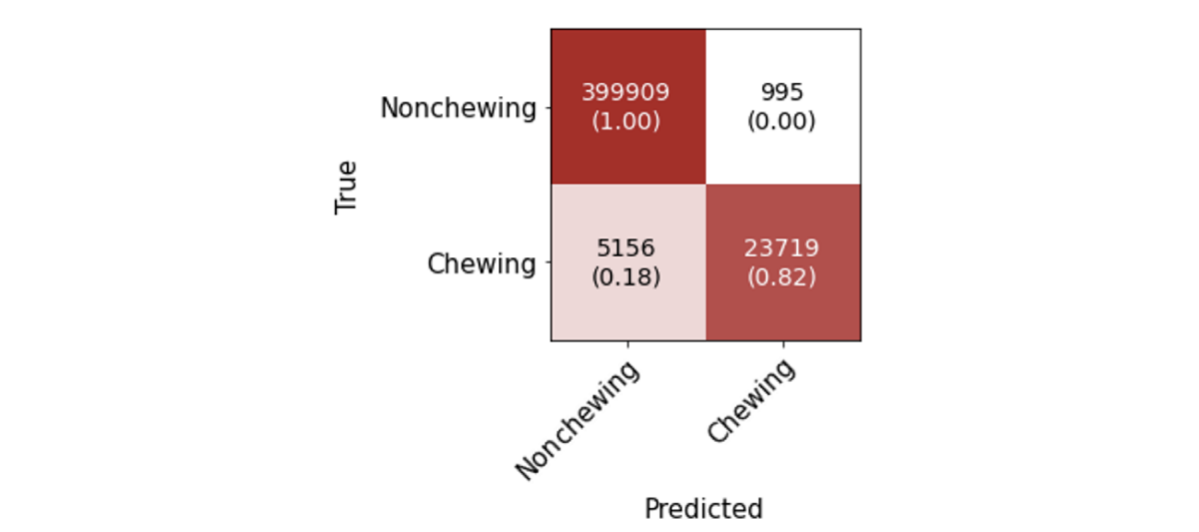
Confusion matrix for the chewing detection model evaluated on the real-life data set on window level.

This evaluation allows us to explore whether a model trained with seminaturalistic behavior data collected in a laboratory setting can perform well on a real-life data from unseen participants. Figure 7 presents the results obtained on the real-life data set at a window level using the model for chewing detection. The classification report is shown in Table 6. It shows that the model achieved precision of 0.95, recall of 0.82, and an *F*_1_-score of 0.88 for the eating class. The accuracy of this model was 98%.

**Figure 8:**
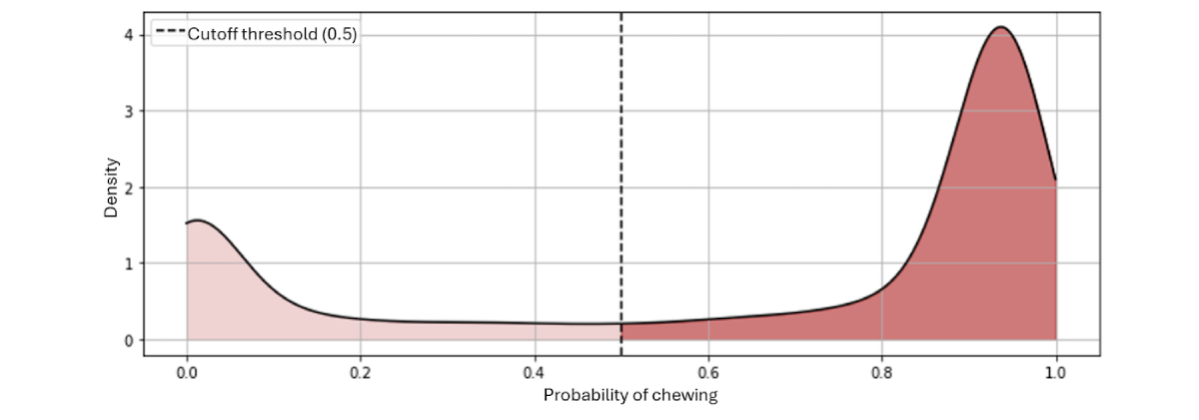
Probability density function of the model’s output probabilities for the chewing-labeled instances.

We derived the probability density function of the model’s probability outputs. The resulting graph, depicted in Figure 8, reveals a bimodal distribution, exhibiting one smaller peak near a probability of 0.2 and a larger, more substantial peak beginning at approximately 0.8 probability. The prominence of the second peak starting from a higher probability threshold signifies the model’s strong confidence in identifying chewing activity, within the labeled eating segments. In addition, the predictions around the first peak can be interpreted as instances where the model is relatively certain that chewing is not occurring within the eating-labeled segments.

These results are in line with our expectations and understanding of the real-life data set. As previously described, the ground truth of the real-life data set contains only the information when eating segments took place. When evaluating the chewing detection method on data set where eating segments are labeled, the presence of false negatives can be attributed to the nature of the data set. Eating segments may encompass various activities beyond just chewing, such as talking, short breaks between bites, holding food, and similar. Therefore, segments labeled as “eating” may indeed involve nonchewing activities.

**Table 6:** Classification report for the chewing detection model evaluated on the real-life data set on window level.

Class	Precision	Recall	*F*_1_-score
Noneating	0.99	1.00	0.99
Eating	0.95	0.82	0.88
Macroaverage	0.97	0.91	0.94

#### Evaluation of the Method for Eating Segment Detection

As previously described, the ground truth for the real-life data set contains information when the eating segments took place. This means that the annotated eating segments may contain short breaks between bites, conversations, food preparation, and similar. Because of this, we evaluated the eating segments detection based on the temporal information of the chewing detection algorithm as described in the *Detection of Eating Segments* section.

The results obtained on a segment level are shown in Table 7. This table contains the total number of eating segments, number of detected eating segments, and falsely detected eating segments for each participant. An eating segment is considered as detected if >50% of the instances in the labeled segment are predicted as chewing. The result of this evaluation shows that from total of 74 eating segments labeled by the participants, we can accurately detect 71 eating segments. The number of the falsely detected eating segments is relatively low for all participants, having total of 7 false detections.

Furthermore, we extended our analysis of the real-life data set to explore the suitability of the sensor data obtained from the smart glasses in-the-wild for capturing more detailed eating-related metrics, beyond only detecting instances of eating. In particular, we aimed to quantify the number of chews and the chewing rate within eating segments, although this method was not subjected to formal evaluation, mainly because of the lack of ground truth in the real‑life data set.

**Table 7:** Evaluation of eating segment (ES) detection on the real-life data set, including total number of ES, number of true detected ES, number of false detected ES, and mean duration of falsely detected ES per participant.

Participant ID	Total ES	True detected ES	False detected ES	Mean duration of falsely detected ES, SD (min)
1	5	4	0	—^a^
2	12	12	1	1.77 (0)
3	20	20	0	—
4	7	6	2	0.72 (0.5)
5	5	4	3	0.54 (0.2)
6	15	15	0	—
7	4	4	1	0.46 (0)
8	5	5	0	—
Total	74	71	7	—

^a^Not available.

#### Estimation of Chewing Rate and Number of Chews Using Real-Life Data

**Figure 9:**
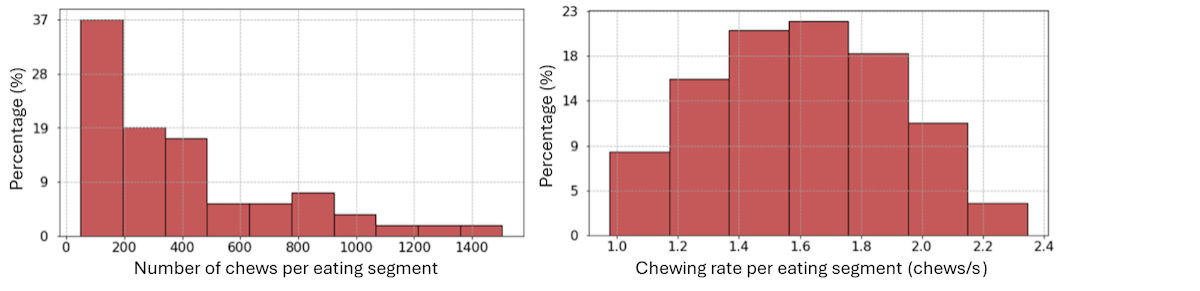
Distribution of estimated number of chews and chewing rates per eating segment in the real-life data set.

After using the previously described methodology for calculating the number of chews on a window level for the eating segments, the resulting values ranged from 48 to 1505. The distribution of these values, as depicted in Figure 9A, indicates that participants have recorded both short snacks and long-duration meals, reflecting the diversity of eating behaviors captured in the data set.

Similarly, upon applying the approach for chewing rate estimation on a window-level for the eating segments, the derived values ranged between 0.8 and 2.3 chews/s. Notably, these values align with expectations observed in real-life eating scenarios [28]. Figure 9B depicts the distribution of the chewing rate values. Figure S6 in Multimedia Appendix 1 presents the mean chewing rate and the total number of chews for all eating segments across all participants in the data set.

## Discussion

### Principal Findings

While most people know that modifying eating behavior is key to sustained weight management, improving what is not measured is difficult. Traditional methods of eating assessment typically summarize dietary measures at the hour, day, week, or even year level [10]. Although these can clarify high-level relationships between eating behavior and disease risk, important short-term patterns are not measured and cannot be explored with the traditional methods. The ability to explore microlevel eating activities—such as meal microstructure (eg, meal duration and chewing frequency) [29] and food choices [30]—is important because they play an important role on food selection, dietary intake, and ultimately, obesity and disease risk [31-33].

In this study, we explored the potential of optical tracking sensors integrated into smart glasses for detection of eating, focusing on chewing activity as a critical component of dietary monitoring. Table 8 compares our study with existing glasses-based eating and chewing monitoring systems, highlighting differences in study goals, sensor types, number of participants, study setups, and performance metrics. It is important to note that the performance metrics may not be directly comparable due to differences in sensors, devices, population, and evaluation setup. Unlike most studies, which primarily monitor eating and drinking behaviors, our approach provides a more granular analysis by specifically detecting chewing activities. Only 2 other studies, including Zhang and Amft [18], have focused on chewing as an activity, and among these, only Zhang and Amft [18] and our study have conducted evaluations in real-world settings. Our results are slightly better, likely because electromyography sensors used by Zhang and Amft [18] require skin contact, whereas our optical sensor-based tracking does not, making our estimates more robust and less intrusive.

Accurate estimates of chewing can further be used for calculating eating rate. Such granularity offers better insights for nutritional management. For example, eating rate has gained interest over recent years, as studies suggest a link between eating quickly and being overweight [31]. Other studies suggest that faster eating rate is associated with higher BMI and higher energy intake [32]. Studies also suggest that eating rate is independently associated with insulin resistance [33], which might be explained by the rapid entrance of glucose into the circulation at the beginning of the meal [34].

Our investigation spanned controlled laboratory settings to the real-life environment, providing a robust assessment of the technology’s effectiveness and practicality. The system leverages contactless optical tracking technology—OCO, to monitor facial muscle activation related. These activations are further processed by a DL model for detection of eating and chewing segments. On the basis of the results of the experiments where we evaluated various DL architectures, ConvLSTM model was selected as the best-performing model for identifying chewing events in our eating detection experiments. Regarding the real-life experiments, our method for chewing detection and eating segments detection, validated on data collected in-the-wild with 8 participants, demonstrates promising results. The model achieved high precision (0.95) and recall (0.82) for the eating class, with an *F*_1_-score of 0.88 at a window level. However, false negatives in chewing detection can be attributed to the diverse activities encompassed within eating segments beyond just chewing, such as talking or short breaks between bites. Evaluating eating segments detection based on temporal information from the chewing detection algorithm revealed accurate detection of 71 out of 74 labeled eating segments, with only 7 false detections across participants.

Regarding the sensor positioning, based on the results in Table 4 and the data from 128 participants, we observed that the cheek sensor outperforms the temple sensor, achieving a recall of 0.88 compared with 0.84 for detecting chewing segments. The addition of temple sensor data shows a modest 1 percentage point improvement in recall, indicating its supplementary role in enhancing overall performance.

Regarding the segmentation window size, as we increased the window size from 2 to 10 seconds, the ConvLSTM model’s precision, recall, and *F*_1_-score improved. However, at a 10-second window, performance plateaued, likely due to the inclusion of irrelevant nonchewing data. Despite better results at 6 and 10 seconds, we chose a 4-second window for its lower computational load and energy consumption. This aspect is crucial for deployment on mobile or wearable devices, where processing power and battery life are limited. Moreover, the use of a 4-second window has proven to offer stable performance that ensures the model is sufficiently fast to adapt to changes in eating behavior without substantial delays, due to the 1-second sliding segment.

**Table 8:** Comparison with glasses-based eating and chewing monitoring systems.

Study	Goal	Sensors	Participants, n	Setup	Performance
Bedri et al [13], 2020	Eating and drinking	IMU^a^, proximity, and camera	18 (laboratory) and 5 (real-world)	Laboratory and real world	*F*_1_-score: 0.89
Shin et al [14], 2022	Eating	Piezoelectric and IMU	30	Real-world	*F*_1_-score: 0.92
Bello et al [15], 2023	Facial expressions and eating and drinking	IMU, pressure, microphone, force, and piezoelectric	10	Real-world	*F*_1_-score: 0.86 (expressions); 0.94 (eating or drinking)
Farooq and Sazonov [16], 2016	Eating and physical activity	Piezoelectric and IMU	10	Laboratory	*F*_1_-score: 99% (eating vs activity)
Chung et al [17], 2017	Chewing and 5 other activities	Load cells	10	Laboratory	*F*_1_-score: 94%
Zhang and Amft [18], 2018	Chewing and eating	EMG^b^ sensors	10	Laboratory and real-world	Precision or recall: 95% (laboratory) and 78% (real-world)
This study	Chewing and eating	OCO optical sensors	128 (laboratory) and 8 (real-world)	Laboratory and real-world	*F*_1_-score: 0.91 (laboratory) and 0.88 (real-world)

^a^IMU: inertial measurement unit.

^b^EMG: electromyography.

### Limitations and Future Work

Regarding limitations, while our study included data from >100 participants, most of the data were collected in controlled setup. In contrast, our real-world data came from 8 participants observed for 2 days. To strengthen these findings, a broader and more prolonged study is needed.

Regarding the technical aspects of the system, the technology should be assessed across various demographics and age groups to ensure its generalizability. Factors such as the shape of a participant’s head or nose might alter sensor position and data quality, and participants’ adherence to wearing the device properly must be considered. Developing personalized models that adapt to individual eating patterns and preferences could improve the system’s accuracy and user acceptance. Machine learning algorithms that learn and adapt to each user’s unique behaviors over time could provide personalized and more accurate monitoring.

In terms of system application domains, combining the eating detection system with nutritional analysis tools could provide a comprehensive solution for monitoring not only eating behaviors but also dietary intake and nutritional quality, offering more actionable insights for health interventions. Furthermore, investigating the long-term impact of using such monitoring systems on health outcomes, including weight management, metabolic health, and behavior change, could provide valuable evidence for the efficacy of these technologies in promoting healthy eating habits and preventing chronic diseases.

### Conclusions

Our study demonstrates the efficacy and feasibility of using optical tracking sensors integrated into smart glasses, particularly with the OCO technology, for noninvasive monitoring of eating behaviors, with a focus on chewing detection and eating segment detection. Through rigorous experimentation on data from 128 in-laboratory participants and 8 real-world participants, we determined that the proposed approach can accurately detect chewing activity in both laboratory and real-life scenario, highlighting the promising potential of this system for dietary monitoring applications.

## Appendix

Multimedia Appendix 1 2D and 3D plots illustrating sensor data during eating, step-by-step predictions of the proposed method, false positive analyses categorized by activity type, and an extended review of related work on wearable technology for eating detection.

## Abbreviations

CNN: convolutional neural network
ConvLSTM: convolutional long short-term memory
DL: deep learning
FLOPS: floating-point operations per second
FP: false positive
HMM: hidden Markov model
LSTM: long short-term memory
