# Supplementary material for: Controlled and Real-Life Investigation of Optical Tracking Sensors in Smart Glasses for Monitoring Eating Behavior Using Deep Learning: Cross-Sectional Study
Source: JMIR Mhealth Uhealth. 2024 Sep 26;12:e59469. doi: 10.2196/59469 (PMC11467608; doi:10.2196/59469)
Supplement: Multimedia Appendix 1 [file mhealth_v12i1e59469_app1.docx]

# Multimedia Appendix

This is a Multimedia Appendix to a full manuscript published in the JMIR mHealth and uHealth. For full copyright and citation information see http://dx.doi.org/10.2196/59469

## Supplemental Figures

Figure S1 illustrates the OCOsense™ glasses and the respective sensor placements, the green rectangles represent the OCO™ sensors that are used in this study.


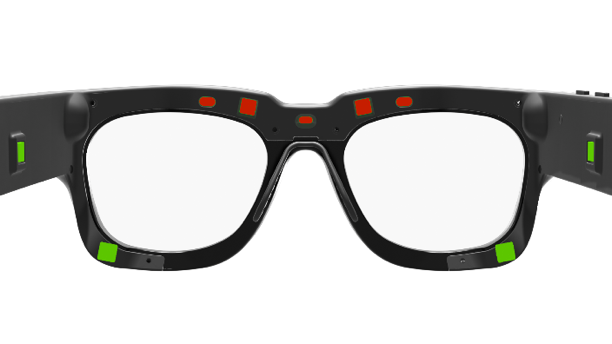

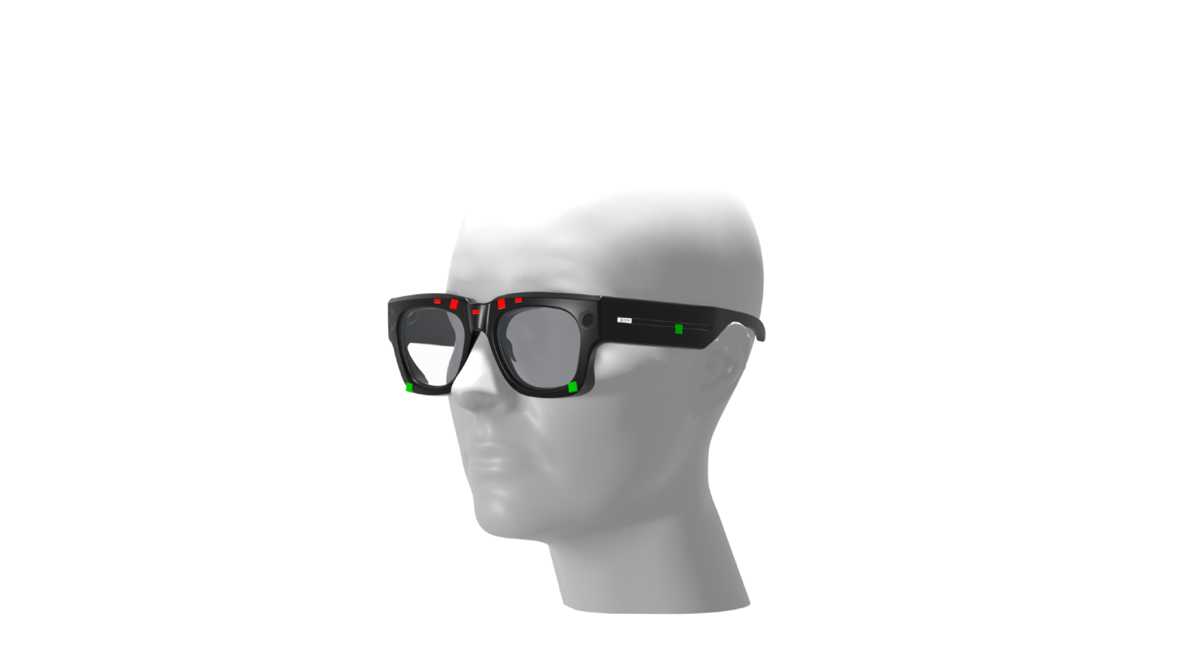


Figure S1. A three-dimensional rendering of OCOsense™ glasses on an average head model, with the OCO™ sensors depicted as red (not used in this study) and green rectangles (used in this study).

Figure S2 presents a sample visualization of the gathered data from a single participant during three bites (and the consequent chewing activity), highlighting signals from the left and right temple and left and right cheek. In the figure, each graph shows three distinct events, with each event representing a single bite. Within these events, there are noticeable variations in amplitude that correspond to the chewing process associated with each bite.


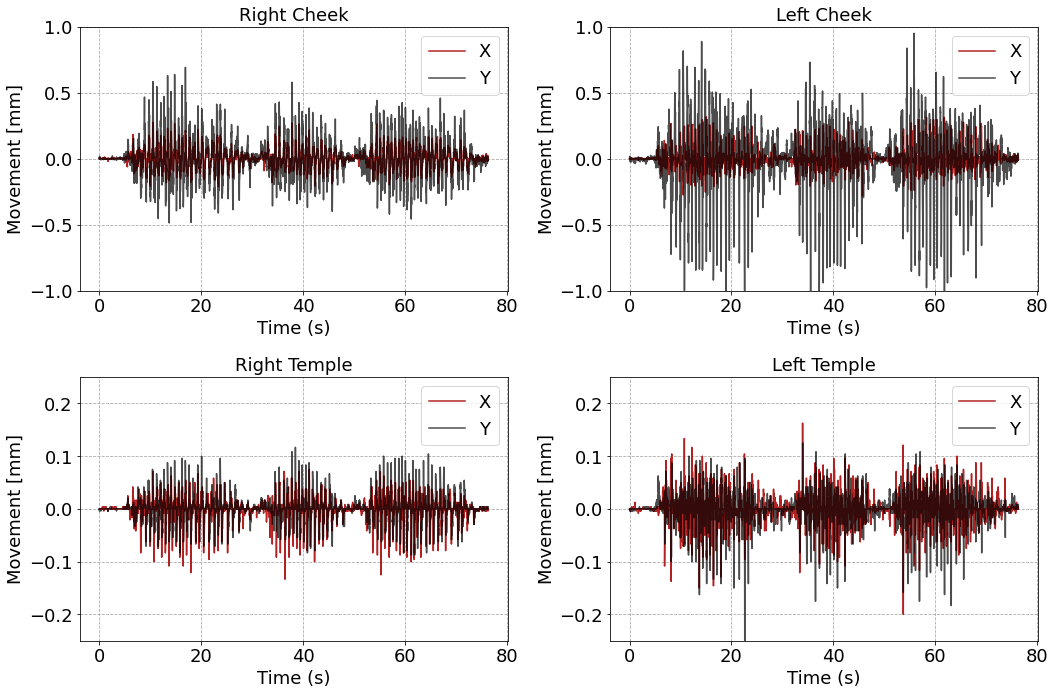


Figure S2. Data from the optical tracking sensors (X and Y) on the left and right cheek and temple recorded during three distinct bites and the corresponding chewing process.

Figure S3 presents a three-dimensional plot of the sensor data captured during a single bite. To create these visualizations, cumulative sum operations were applied to the *X* and *Y* signals from the cheek and temple sensors. These plots offer an insight into the dynamics of chewing, showing patterns in the sensor data corresponding to specific phases of the chewing process. At the beginning, we can observe minimal fluctuation in the sensor data. This indicates the period when the participant has not begun to chew, with the cheek and temple sensors registering only minor movements. Once chewing begins, a clear pattern of circular movements appears in the plots, corresponding to the repetitive nature of chewing. This shift is seen as an increase in the amplitude of the cheek sensor signals, aligning with the activation of the muscles responsible for chewing. Although the temple sensor signals show smaller amplitude, they still pick up a similar circular pattern that reflects the movements related to chewing. As the chewing sequence nears its end, the amplitudes in the sensor readings gradually decrease, reflecting a decrease in the intensity of muscle activity as the individual completes the chew.


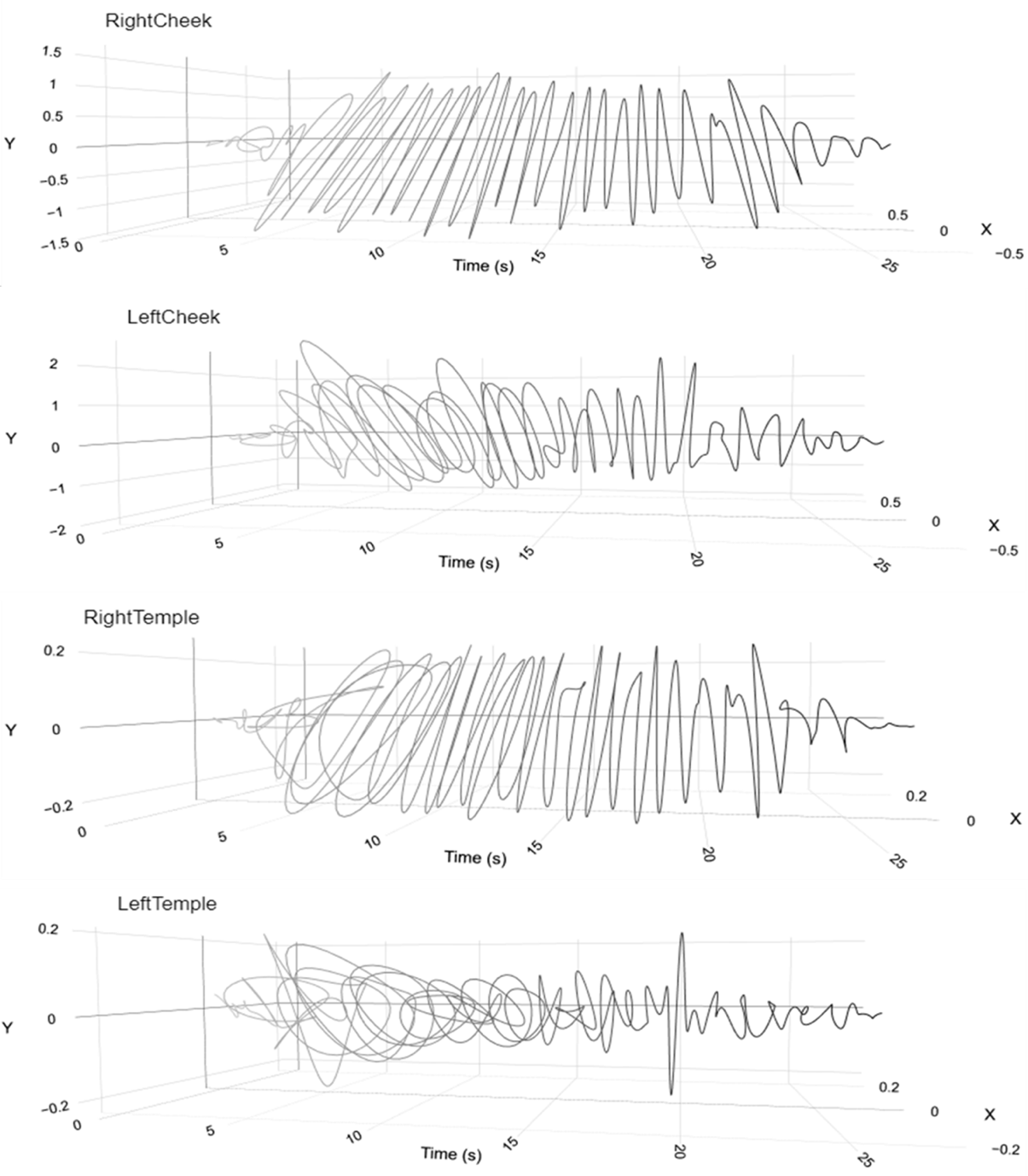


Figure S3. Three-dimensional visualization of cheek and temple sensor data during a single bite, revealing distinct phases of the chewing process.

Figure S4 illustrates our approach to addressing the temporal dependence between chewing events in real life. We integrate an HMM as a supplementary model to analyze the detected chews from the DL model.


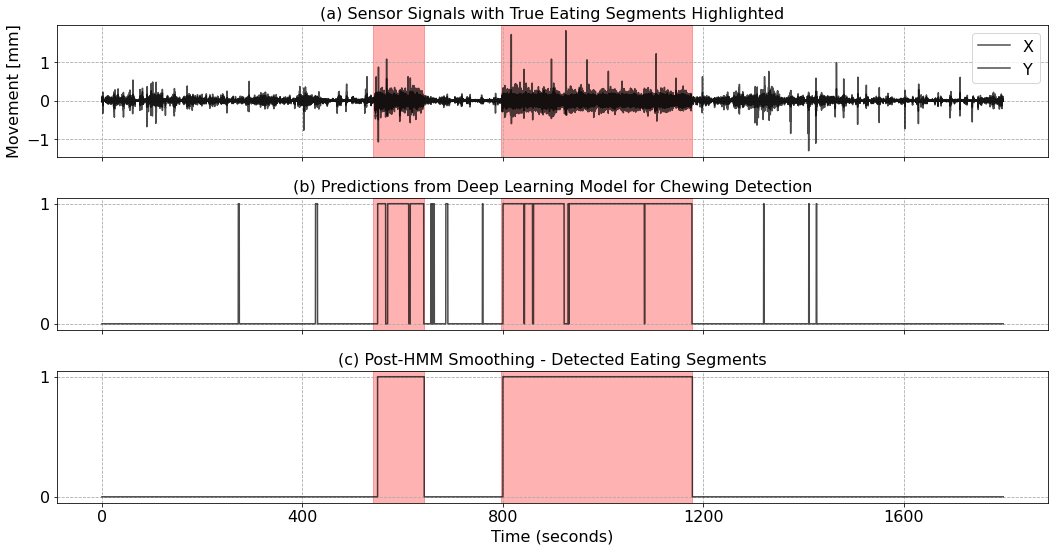


Figure S4. Eating Segments Detection Process: (a) Raw data from a cheek sensor with labelled eating segments highlighted in red; (b) Output of the deep learning-based chewing detection model trained using data from the temple and cheek sensor positions, with value of one representing detected chewing activity. The highlighted regions in red represent the ground truth of the eating segments (c) Output from temporal postprocessing of detected chews using Hidden Markov Model, where value of one represents the detected eating segments.

Figure S5 provides an overview of the false-positive rates for various non-eating activities detected by the ConvLSTM model. Socializing has the highest false-positive rate (0.72%), followed by reading at around 0.28%. These two activities involve speaking, and it is expected that they can sometimes be confused with eating due to similar facial movements.


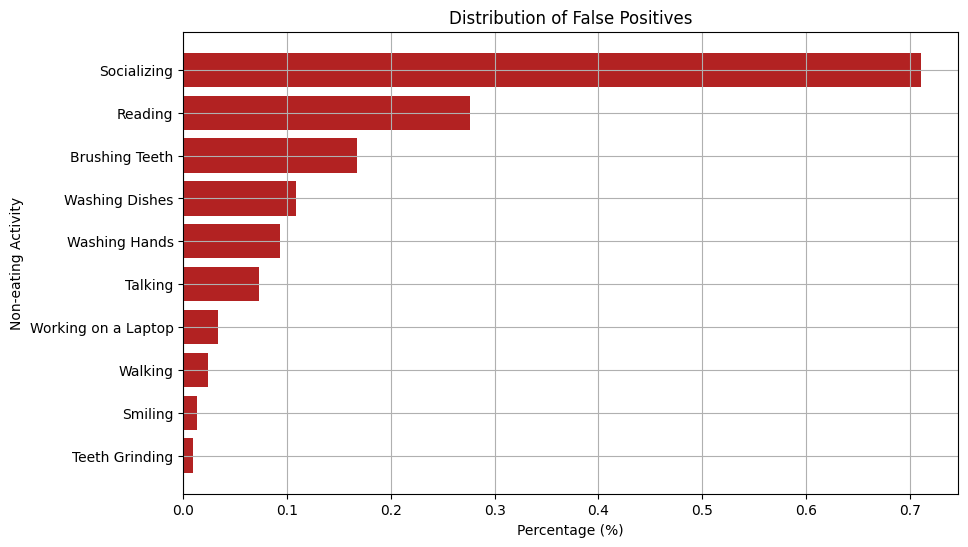


Figure S5. False-positive rates for the non-eating activities. ConvLSTM model.

In Figure S6, we present the mean chewing rate and the total number of chews for all eating segments across all participants in the real-life dataset. Each participant is represented on the x-axis. The number of chews for the eating segment is represented by the size of the circles, with each circle symbolizing a separate eating segment. The smaller the circle, the smaller the number of chews associated with that particular segment. Meanwhile, the mean chewing rate is indicated by the colour of the circle. From the analysis, it's evident that each participant maintains relatively similar chewing rate across their meals. Participant 3 consistently exhibits the lowest chewing rate compared to other participants.


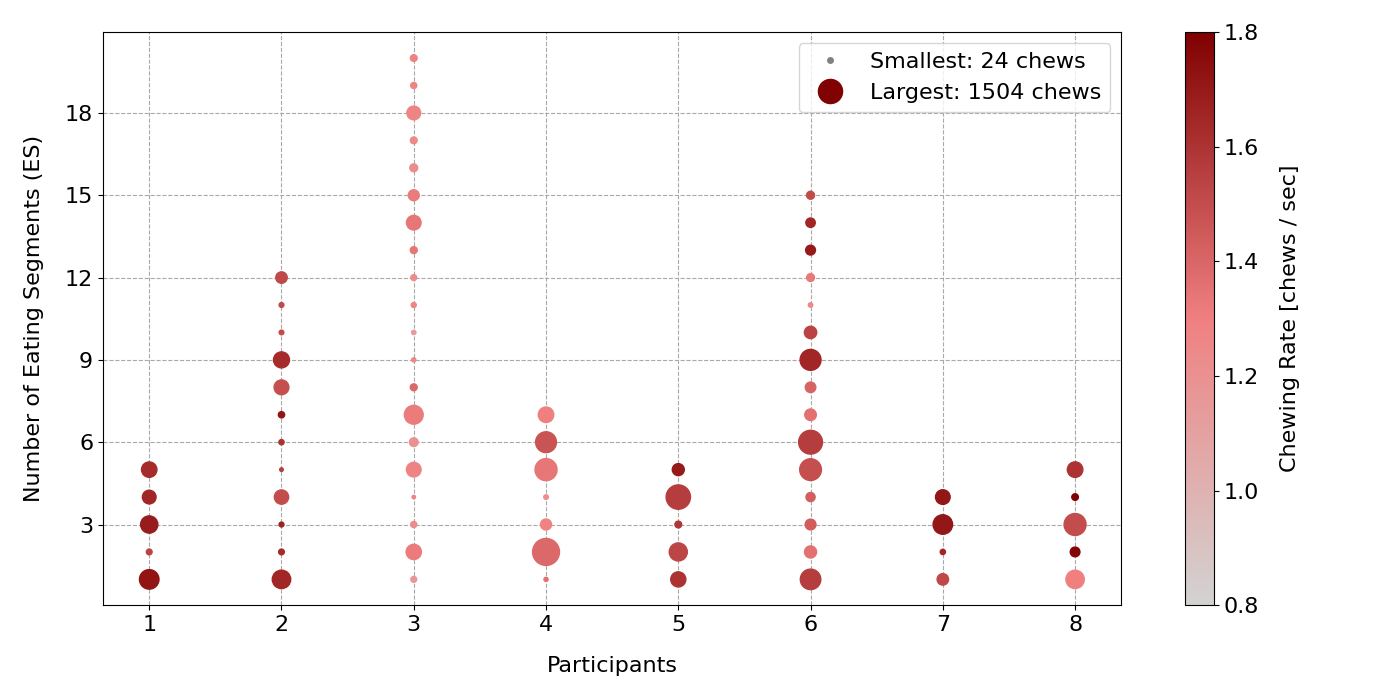


Figure S6. Mean Chewing Rate and Total Number of Chews per Eating Segment (ES).

## 2. Related work analysis

Over the last ten years, various technologies have been designed to improve the accuracy of food diaries by automatically monitor food consumption. Numerous devices, particularly smartphones have been employed to reduce user effort compared to manual systems. However, wearable devices (smartwatches and smart glasses) are the most promising approaches. These typically employ sensors, including accelerometers, gyroscopes, magnetometers, and cameras, to identify actions related to eating, e.g., bringing food to the mouth, chewing, and swallowing. Importantly, the majority of previous studies were carried out in controlled environments, thus real-life studies still remain under explored [1, 2, 3]. That is because creating, implementing, and evaluating automatic eating trackers involve expensive user studies, privacy sensitive data, and dealing with unreliable sensor data and ground truth. From a technical perspective, a complex challenge lies also in the creation of tools (e.g., machine‑learning models) that can accurately distinguish between eating-related actions and similar behaviours (e.g., speaking or teeth clenching).

The section provides a more detailed overview of the related work studies. In the first subsection, we provide a wider overview of wearable sensors for monitoring eating activities. In the second subsection, we present glasses-based systems for monitoring facial activities other than eating (e.g., facial expression and gesture recognition). In the third subsection, we focus on studies that are closest to ours, i.e., glasses-based systems for monitoring eating activities.

#### 2.1 Wearable sensors for monitoring eating activities

For a detailed related work analysis on wearable sensors for eating detection we refer the reader to a recent scoping review by Bell et al. [3]. On average, the studies deployed more than two wearable sensors per study, including accelerometers, gyroscopes, microphones, piezoelectric sensors, radio frequency (RF) signals, smartwatches, cameras, electromyography (EMG) electrodes, and infrared proximity sensors. Most studies utilized accelerometers (i.e., 25 out of 40 studies) and/or gyroscopes (i.e., 15 out of 40 studies), typically embedded within a wrist-worn device [10]. The user study sample sizes ranged from 1 to 104 participants. Regarding ground truth, data was gathered through self-reports including mobile device‑assisted ecological momentary assessments (mEMAs), objective methods (e.g., wearable video cameras), or some combination of the three. For example, some studies had participants use logs, diaries, or even video cameras to record eating episodes, while others received real-time messages to confirm eating gestures detected by the sensors. The objective in most of the studies was to develop systems that can detect eating events (i.e., 37 out of 40 studies). Out of the 40 studies, only three studies focused on estimating chewing activities and only one study focused on detecting hand-to-mouth gestures.

#### 2.2 Glasses-based wearables for monitoring facial activities

Glasses-based sensing has been popularized in recent years. Typical use-cases involve facial expression and gestures recognition [11, 12, 13, 14], cognitive workload estimation [15], and other tasks for improving human-computer interaction. For example, Meyer et al. [15] combined a commercial eye-tracker with an IMU in a glasses-based wearable to detect seven physical or cognitive tasks (talk, solve, read, watch a video, type, walk, and cycle). Sato et al. [11] and Gjoreski et al. [12] investigated devices with EMG sensors positioned over specific facial muscles to estimate facial expressions and affect. Electrooculography (EOG) sensing has been utilized in several studies [13, 14, 16]. Yeo et al. presented JINSense smart glasses for gesture recognition and human-computer interaction [13]. Li et al. developed KissGlass, a smart-glasses-based method for monitoring kinds of cheek kissing gestures [16]. Rostaminia et al. [14] developed W!NCE, a smart-glasses-based method for monitoring upper-face activation (brow movement, cheek movement, nose wrinkling, and blinking). In addition to the EMG- and EOG-based approaches, studies have explored smart glasses in combination with camera-based sensing [17] and capacitive sensing [18] for facial expressions and head gestures recognition.

#### 2.3 Glasses-based wearables for monitoring eating activities

Regarding the studies on glasses-based solutions for monitoring eating activities, Bedri et al. [4] combined inertial and proximity sensors to detect food intake events. The system also uses an integrated a camera to capture pictures of the food as the user consumes it. The system preprocesses the sensor data to filter out noise and normalize signals. Feature extraction follows, where specific characteristics that indicate eating or drinking actions are identified. Machine learning algorithms analyse these features to classify the actions as eating, drinking, or non-eating/drinking activities. The system was evaluated in two studies involving 23 participants overall, under semi-constrained and unconstrained settings. FitByte achieved an average F1-score of 89% in detecting eating and drinking episodes.

Shin et al. [5] presented MyDJ, an eating detection system designed to attach to an eyeglasses frame, utilizing a piezoelectric and accelerometer sensors. The system aims to monitor food intake in uncontrolled environments while being energy efficient. The system preprocesses the sensor data, then features are extracted, and then machine learning models are utilised to classify the sensor data as eating or non-eating events. In a user study involving 30 participants in real-world conditions, MyDJ demonstrated a 91.9% F1-score in detecting eating episodes.

MeciFace [6], is a system that uses glasses equipped with inertial and mechanomyography sensors to monitor facial and eating activities in real time. The system aims to provide an energy-efficient and privacy-aware tool for monitoring facial activities. The processing pipeline involves multimodal sensor fusion, employing lightweight neural networks for real-time edge recognition. The system was evaluated in a user study with five volunteers in natural eating settings, achieving an F1-score of 90% for detecting eating and drinking events.

Chung et al. [8] presented a device that uses load cells embedded in eyeglass hinges to monitor temporalis muscle activity. The processing pipeline involves signal preprocessing, feature extraction from temporal and spectral domains, and classification using a Support Vector Machine (SVM) with a Radial Basis Function (RBF) kernel. The study was conducted in controlled environments with 10 participants, testing six activities: natural head movement, left chewing, right chewing, left wink, right wink, and talking. The system achieved an average F1-score of 94% for differentiating between the six activities.

Zhang et al. [9] presented smart glasses for chewing and eating detection in both controlled and free-living environments using integrated EMG sensors. The system processing involves EMG signal filtering, chewing cycle detection, and eating event identification through chewing frequency. A study with 10 participants demonstrated the system's efficacy, showing precision and recall near 78% in real-world settings and around 95% in controlled settings for chewing detection. Additionally, it achieved a 94% accuracy in classifying food hardness during a controlled experiment (e.g., the system classifies banana vs. cucumber vs. carrot).

Farooq and Sazonov [7] presented an approach integrating a piezoelectric strain sensor and accelerometer onto eyeglass temples for detecting food intake and physical activity. They aimed to monitor eating behaviours events in active conditions. The processing pipeline involved signal filtering, feature extraction, and multiclass classification using sensor fusion and a two-stage classification strategy. In a user-study with 10 participants in a laboratory setting, the approach demonstrated high efficacy with a 99.85% F1-score in distinguishing eating actions from physical activities.

## References

1. Vu T, Lin F, Alshurafa N, Xu W. Wearable Food Intake Monitoring Technologies: A Comprehensive Review. *Computers*. 2017; 6(1):4. https://doi.org/10.3390/computers6010004
2. Heydarian H, Adam M, Burrows T, Collins C, Rollo ME. Assessing Eating Behaviour Using Upper Limb Mounted Motion Sensors: A Systematic Review. *Nutrients*. 2019;11(5):1168. Published 2019 May 24. doi:10.3390/nu11051168
3. Bell BM, Alam R, Alshurafa N, et al. Automatic, wearable-based, in-field eating detection approaches for public health research: a scoping review. *NPJ Digit Med*. 2020;3:38. Published 2020 Mar 13. doi:10.1038/s41746-020-0246-2
4. Bedri A, Li D, Khurana R, Bhuwalka K, Goel M. FitByte: Automatic Diet Monitoring in Unconstrained Situations Using Multimodal Sensing on Eyeglasses. In: Proceedings of the 2020 CHI Conference on Human Factors in Computing Systems (CHI '20). New York, NY: Association for Computing Machinery; 2020:1-12. doi:10.1145/3313831.3376869
5. Shin J, Lee S, Gong T, Yoon H, Roh H, Bianchi A, Lee S-J. MyDJ: Sensing Food Intakes with an Attachable on Your Eyeglass Frame. In: Proceedings of the 2022 CHI Conference on Human Factors in Computing Systems (CHI '22). New York, NY: Association for Computing Machinery; 2022. 341, 1-17. doi:10.1145/3491102.3502041
6. Bello H, Suh S, Zhou B, Lukowicz P. MeciFace: Mechanomyography and Inertial Fusion based Glasses for Edge Real-Time Recognition of Facial and Eating Activities. arXiv. Preprint posted online June 23, 2023. doi: 10.48550/arXiv.2306.13674
7. Farooq M, Sazonov E. A Novel Wearable Device for Food Intake and Physical Activity Recognition. *Sensors (Basel)*. 2016;16(7):1067. Published 2016 Jul 11. doi:10.3390/s16071067
8. Chung J, Chung J, Oh W, Yoo Y, Lee WG, Bang H. A glasses-type wearable device for monitoring the patterns of food intake and facial activity. *Sci Rep*. 2017;7:41690. Published 2017 Jan 30. doi:10.1038/srep41690
9. Zhang R, Amft O. Monitoring Chewing and Eating in Free-Living Using Smart Eyeglasses. *IEEE J Biomed Health Inform*. 2018;22(1):23-32. doi:10.1109/JBHI.2017.2698523
10. Thomaz E, Essa I, Abowd GD. A Practical Approach for Recognizing Eating Moments with Wrist-Mounted Inertial Sensing. *Proc ACM Int Conf Ubiquitous Comput*. 2015;2015:1029-1040. doi:10.1145/2750858.2807545
11. Sato W, Murata K, Uraoka Y, Shibata K, Yoshikawa S, Furuta M. Emotional valence sensing using a wearable facial EMG device. *Scientific Reports*. 2021;11(1). doi:10.1038/s41598-021-85163-z
12. Gjoreski M, Kiprijanovska I, Stankoski S, et al. Facial EMG sensing for monitoring affect using a wearable device. *Sci Rep*. 2022;12(1):16876. Published 2022 Oct 7. doi:10.1038/s41598-022-21456-1
13. Yeo HS, Lee J, Woo W, Koike H, Quigley AJ, Kunze K. JINSense: Repurposing Electrooculography Sensors on Smart Glass for Midair Gesture and Context Sensing. Published online May 8, 2021. doi:10.1145/3411763.3451741
14. Rostaminia S, Lamson A, Maji S, Rahman T, Ganesan D. W!NCE. *Proceedings of the ACM on Interactive, Mobile, Wearable and Ubiquitous Technologies*. 2019;3(1):1-26. doi:10.1145/3314410
15. Meyer J, Frank A, Schlebusch T, Kasneci E. U-HAR. *Proceedings of the ACM on Human-Computer Interaction*. 2022;6(ETRA):1-19. doi:10.1145/3530884
16. Li R, Lee J, Woo W, Starner T. KissGlass. *Proceedings of the Augmented Humans International Conference*. Published online March 16, 2020. doi:10.1145/3384657.3384801
17. Yan Z, Wu Y, Zhang Y, Chen X ’Anthony’. EmoGlass: an End-to-End AI-Enabled Wearable Platform for Enhancing Self-Awareness of Emotional Health. *CHI Conference on Human Factors in Computing Systems*. Published online April 27, 2022. doi:10.1145/3491102.3501925
18. Matthies DJC, Weerasinghe C, Urban B, Nanayakkara S. CapGlasses: Untethered Capacitive Sensing with Smart Glasses. *Augmented Humans Conference 2021*. Published online February 22, 2021. doi:10.1145/3458709.3458945
